# The moderating effects of sex, age, and education on the outcome of combined cognitive training and transcranial electrical stimulation in older adults

**DOI:** 10.3389/fpsyg.2023.1243099

**Published:** 2023-09-22

**Authors:** Christine Krebs, Jessica Peter, Esther Brill, Stefan Klöppel, Anna-Katharine Brem

**Affiliations:** ^1^University Hospital of Old Age Psychiatry and Psychotherapy, University of Bern, Bern, Switzerland; ^2^Graduate School for Health Sciences, University of Bern, Bern, Switzerland; ^3^Department of Old Age Psychiatry, Institute of Psychiatry, Psychology and Neuroscience, King’s College London, London, United Kingdom

**Keywords:** older adults, tDCS, tACS, cognitive training, education, age, sex

## Abstract

Computerized cognitive training (CCT) has been shown to improve cognition in older adults via targeted exercises for single or multiple cognitive domains. Combining CCT with non-invasive brain stimulation is thought to be even more effective due to synergistic effects in the targeted brain areas and networks. However, little is known about the moderating effects of sex, age, and education on cognitive outcomes. Here, we investigated these factors in a randomized, double-blind study in which we administered CCT either combined with transcranial direct (tDCS), alternating (tACS) current stimulation or sham stimulation. 59 healthy older participants (mean age 71.7 ± 6.1) received either tDCS (2 mA), tACS (5 Hz), or sham stimulation over the left dorsolateral prefrontal cortex during the first 20 min of a CCT (10 sessions, 50 min, twice weekly). Before and after the complete cognitive intervention, a neuropsychological assessment was performed, and the test scores were summarized in a composite score. Our results showed a significant three-way interaction between age, years of education, and stimulation technique (*F*_(6,52)_ = 5.53, *p* = 0.007), indicating that the oldest participants with more years of education particularly benefitted from tDCS compared to the sham group, while in the tACS group the youngest participants with less years of education benefit more from the stimulation. These results emphasize the importance of further investigating and taking into account sex, age, and education as moderating factors in the development of individualized stimulation protocols.

**Clinical Trial Registration:**

ClinicalTrials.gov, identifier NCT03475446.

## 1. Introduction

Transcranial electrical stimulation (tES) has been used in various studies to improve cognitive performance in healthy participants and diverse patient populations (Yavari et al., 2018).

Transcranial direct current stimulation (tDCS) is one type of tES which has been frequently used in single sessions or repeatedly with and without concurrent tasks. Today, tDCS is mostly combined with a concurrent task to benefit from synergistic effects of stimulation and intrinsic brain activity (Indahlastari et al., 2021). When tDCS is applied repeatedly with cognitive stimulation in the course of a cognitive intervention the outcomes were promising in cognitive domains like working memory and cognitive control (Elmasry et al., 2015). A recent study reported contrary effects of anodal tDCS in middle aged (50–64 years) and older (65–81 years) adults. While older adults showed better recognition performance after stimulation over the left dorsolateral prefrontal cortex (DLPFC) during encoding, middle aged adults performed worse (Bagattini et al., 2023). Computerized cognitive training (CCT) of working memory and concurrent stimulation moreover benefitted older adults more than young adults (Pergher et al., 2022). Similarly, when comparing younger-old and older-old participants in a combined working memory training and tDCS study, Assecondi et al. (2022) found that older-old with lower working memory capacity profited more from tDCS during working memory training, whereas younger-old with high working memory scores performed significantly better without concurrent tDCS. Age-related brain changes, namely atrophy, lead to an increase in cerebrospinal fluid volume, which in turn affects the direction and the strength of the electrical field reaching the targeted region of interest (Bhattacharjee et al., 2022). For tDCS, this might indicate that stronger currents have to be applied to achieve stimulation effects in older adults. On the other hand, changes in the neurotransmitter system in older adults might increase the efficacy of tDCS even when neuroplasticity decreases over the lifespan (Habich et al., 2020). Despite general age-related brain changes there exist large differences on the individual level caused by environmental and genetic factors (Franke and Gaser, 2019). While this variability supports the inclusion of age as moderating factor in the analysis of tES effects, we were not able to identify such effects in our study combining different tES protocols and CCT (Krebs et al., 2021). Notably, age-related brain changes differ between females and males, pointing out sex as another factor moderating brain stimulation outcomes (Bhattacharjee et al., 2022). However, there are tDCS studies not reporting any sex differences in older adults, see Hayek et al. (2021) for example. Finally, years of education might be another moderating factor for stimulation outcomes. For example, Berryhill and Jones (2012) found that only healthy older adults with more years of education benefitted from stimulation during a working memory task. Years of education are a common proxy of cognitive reserve, which can also be estimated by questionnaires like the cognitive reserve index questionnaire (Nucci et al., 2012). In mild cognitive impairment, we found in a previous study that higher cognitive reserve was associated with stronger tDCS effects, similar as has been reported in the study by Berryhill and Jones (2012), while in Alzheimer’s dementia reverse findings were reported in an episodic memory task (i.e., stronger tDCS effects in individuals with low cognitive reserve) (Krebs et al., 2020). Different approaches, i.e., investigating both education as well as cognitive reserve, might help to elucidate moderating effects differently in various populations. Overall, the results across studies show a large heterogeneity. Apart from differences in study design it seems that also inter-individual differences moderate the efficacy of tDCS, for example age, baseline cognition, years of education, and sex (Koo et al., 2023).

Another tES technique is transcranial alternating current (tACS) stimulation, which involves applying alternating electrical currents in sinusoidal waves at certain frequencies. By targeting specific frequencies, tACS aims to adapt intrinsic brain oscillations and hereby influence cognitive and behavioral functions (Antal and Herrmann, 2016). Stimulation at theta frequency (4–8 Hz) appears to be beneficial for several cognitive processes (Antal and Herrmann, 2016; Antonenko et al., 2016) and gamma tACS (*ca.* 40 Hz) seems to play a crucial role in memory processes and appears to be a promising avenue to alleviate memory impairments in dementia (Manippa et al., 2023). To date, tACS has only rarely been used in combination with CCT. In healthy older adults CCT combined with theta tACS did not result in improvements in multitasking performance on the group level. However, there was a high inter-individual variability indicating that there are likely additional factors at play such as baseline peak theta frequency (Zanto et al., 2021). Another study found that higher age was beneficial when theta tACS was applied during an associative memory task compared to a single session of sham stimulation (Klink et al., 2020). In older adults with mild cognitive impairment a single session of gamma tACS (40 Hz) was more beneficial for executive functions (as assessed with the Stroop task and the Trail-Making-Test) than tDCS (Kim et al., 2021). Grover et al. (2022) found improvements in working memory, after stimulating the prefrontal (gamma tACS) versus the parietal cortex (theta tACS) for 4 days in older healthy adults with effects lasting for 1 month. Notably, participants with lower baseline cognitive functions improved more. Exploratory analysis furthermore revealed stronger effects in males than females, but after correcting for multiple comparisons this finding did not hold. Another study reported a beneficial effect of gamma tACS on episodic memory in subjects with Alzheimer’s disease, but no effect of cognitive reserve as measured with the cognitive reserve index questionnaire (Benussi et al., 2022). Finally, a recent meta-analysis supports positive findings for several cognitive functions and emphasizes stronger effects after offline compared to online tACS (Grover et al., 2023). In young adults, CCT of executive functions (working memory, inhibitory control, cognitive flexibility) in combination with multifocal gamma tACS (40 Hz) did not show an improvement in fluid intelligence (Brem et al., 2018). Another study in young adults showed no sex differences when alpha tACS was applied at the individual alpha frequency on the performance in a mental rotation task, while a significant interaction between stimulation group and sex was found for fluid intelligence (Kasten and Herrmann, 2017). Further analysis showed a trend (*p* < 0.09) for a negative effect of tACS in the individual alpha frequency on fluid intelligence in males (Pahor and Jaušovec, 2016).

Our aim was to investigate the moderating effects of age, years of education, and sex alone as well as their interactions in a cognitive intervention combining CCT with tDCS, tACS, or sham stimulation. Based on previous studies, we hypothesised that stimulation effects would be stronger in individuals with more years of education. We moreover investigated possible moderating effects of cognitive reserve on stimulation outcomes to investigate if potential moderating effects of education on stimulation can be confirmed with this measure. For sex, the limited amount of data, which was moreover mostly collected in young adults, prevented the formulation of a directed hypothesis. Despite our previous nil-findings regarding an effect of age on stimulation outcomes (Krebs et al., 2021), we hypothesised significant effects when considering more complex relationships (i.e., between age and sex or age and education).

## 2. Methods

### 2.1. Study design and participants

**Figure 1:**
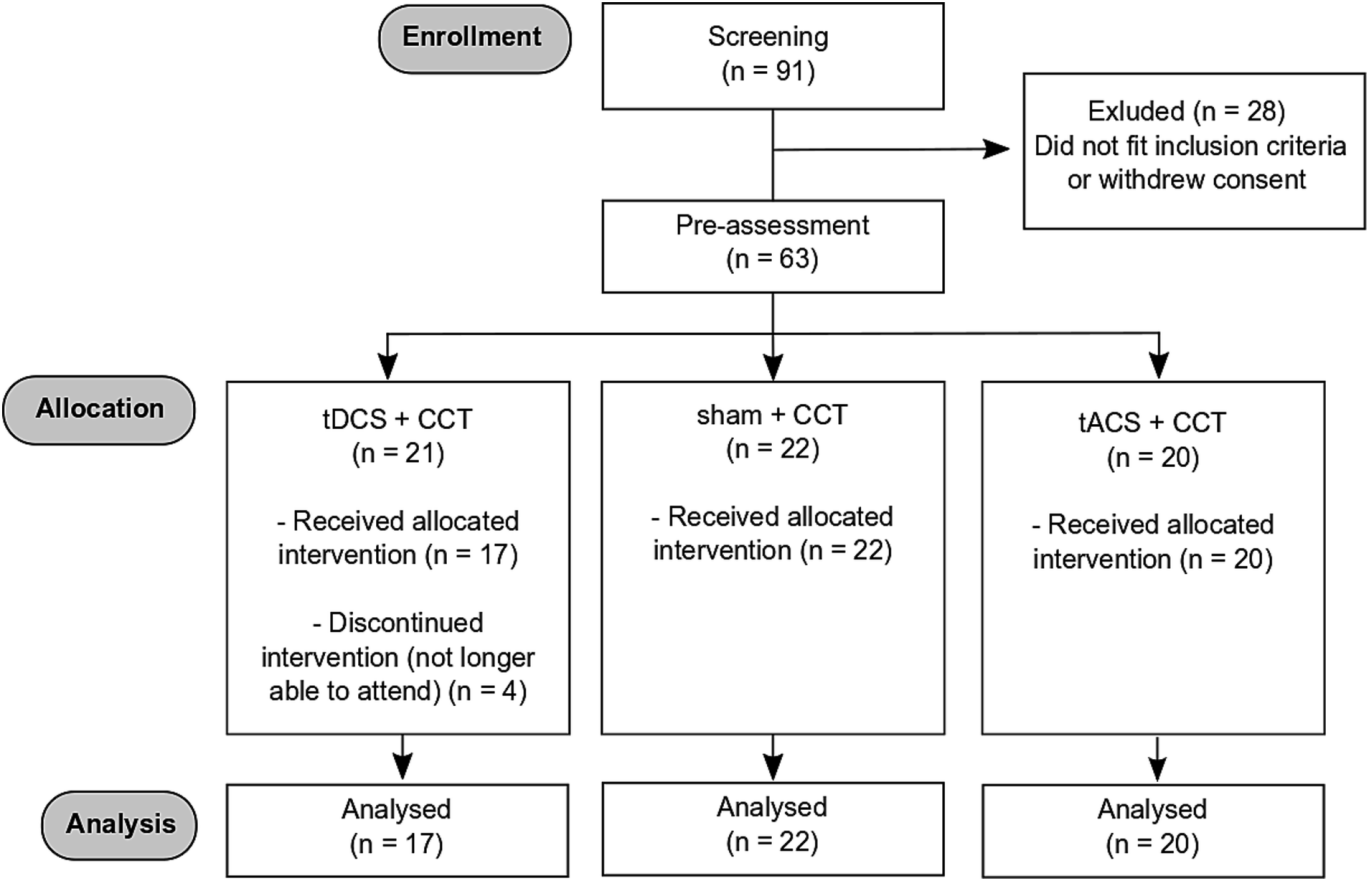
CONSORT flowchart.

The present study was part of a larger study investigating the effect of CCT combined with transcranial electrical stimulation in a double-blind, sham-controlled, and parallel group design (Krebs et al., 2021). Participants were randomly assigned to one of the three stimulation conditions (tDCS, tACS, or sham) prior to their first on-site visit. The final sample contained 59 healthy older participants (mean age 71.7 ± 6.1, range: 61–85; 31 male; years of education median: 14, range: 9–25; see Figure 1 for a flow diagram of participants).

The eligibility criteria were the following: healthy participants (based on self-reports aged between 60 and 85 years, native or fluent German speaker, normal or corrected to normal vision and hearing, and written informed consent). The exclusion criteria were: any history of seizure or stroke, traumatic brain injury, current psychiatric or neurological disorders, substance abuse, metal implants in the head, pacemaker, smoking, psychotropic medication, severe tinnitus and self-reported left-handedness. The study was approved by the local ethics committee (Nr. 2017-02056) and performed in accordance with the Declaration of Helsinki and registered on ClinicalTrials.gov (NCT03475446). Participants gave their written informed consent before study onset.

### 2.2. Cognitive assessments and questionnaires

The cognitive assessment was performed at baseline (i.e., within 6 weeks before intervention onset) and repeated within 2 weeks after the cognitive intervention (except for three participants for whom the delay between the last training and the post-assessment was more than 30 days due to vacations or illness). We used the computerized Vienna Test System (Schuhfried GmbH, Mödling, Austria) to assess verbal and non-verbal memory functions (auditory word list learning: learning sum, delayed recall, d prime (Pallier, 2002), word recognition; continuous figural recognition: d prime), attention functions (divided and selective attention: d prime), and executive functions (inhibition: d prime Go/NoGo; semantic/lexical fluency: total number of words; working memory: block span backwards). Baseline motivation was assessed with the objective achievement motivation test (Brandstätter, 2005). Further executive and attention functions were assessed with paper-pencil tests [5-point test: number of unique designs (Regard et al., 1982); number connection test: average time (Oswald and Roth, 1987)]. Parallel test versions were used whenever available (i.e., MoCA, auditory wordlist learning, fluency, and number connection tests). The primary cognitive outcome was a cognitive composite score that was based on principal component analysis on test scores from the pre-assessment. All scores except the inhibition test scores were included in this cognitive composite score [see Krebs et al. (2021) for single test scores]. To build the composite score, individual raw test scores were scaled to the respective test score from the baseline assessment and then the mean across tests from one time point (i.e., test scores from pre and post assessment) was calculated to calculate the final composite score. Furthermore, participants completed the cognitive reserve index questionnaire (CRIq) (Nucci et al., 2012) and the MoCA.

### 2.3. Intervention: computerized cognitive training combined with non-invasive brain stimulation

**Figure 2:**
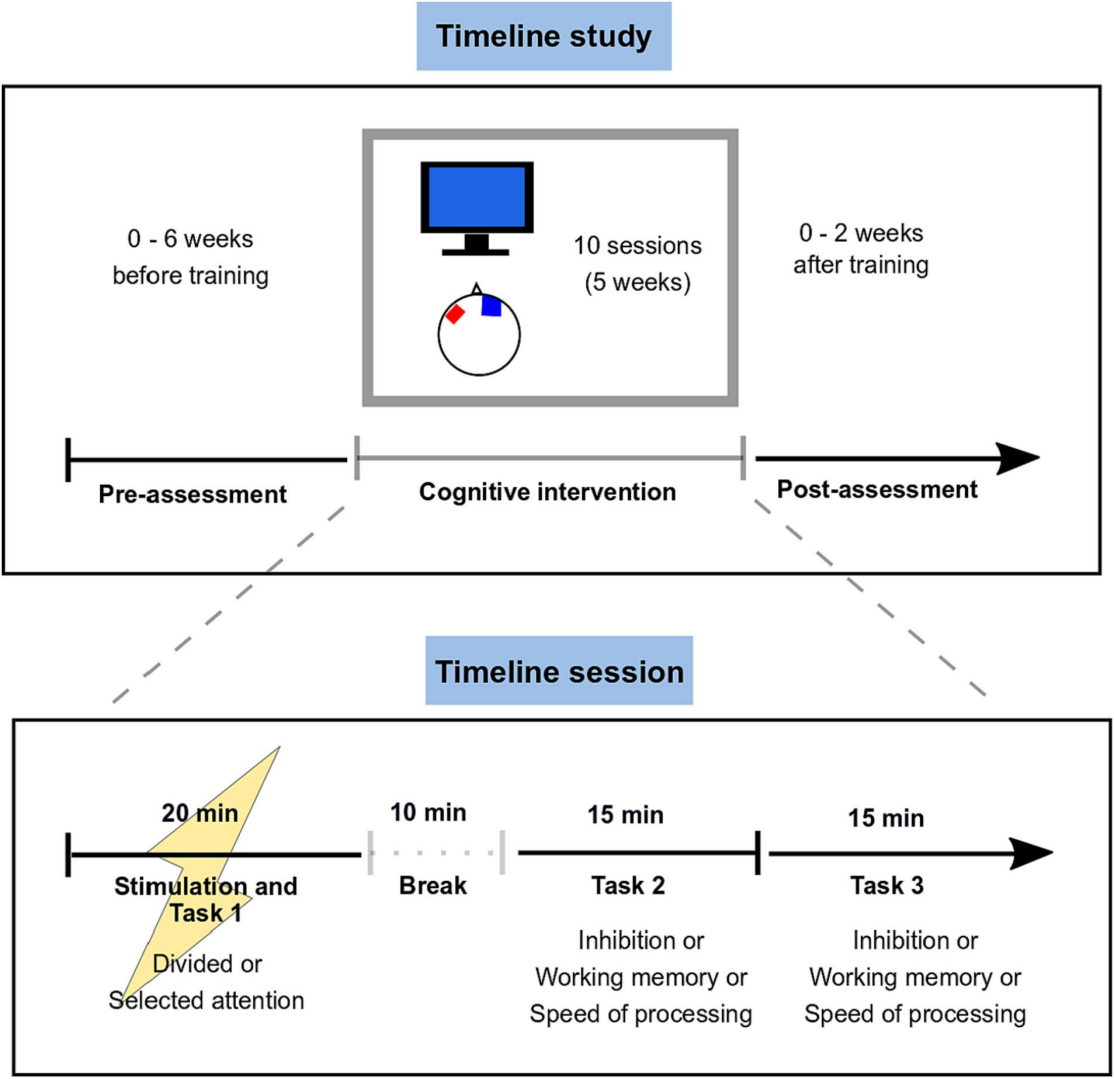
Study design. Participants underwent the pre-assessment (neuropsychological test battery and questionnaires) within 6 weeks before the intervention and completed the post-assessment within 2 weeks after the end of the intervention. During each of the 10 sessions, the CCT was combined with either tDCS, tACS, or sham stimulation during the first 20 min of the trainings. After a 10 min break and removal of the stimulation electrodes, they continued the cognitive training with two further tasks targeting one of three cognitive domains (inhibition, spatial working memory, processing speed).

The intervention consisted of CCT (10 sessions, 50 min, twice weekly at least 2 days apart) combined with either tDCS (2 mA), theta tACS (1 mA, 5 Hz, 0° initial phase shift), or sham stimulation during the first 20 min of each CCT session (DC-Stimulator PLUS, Neuro-Conn GmbH, Ilmenau, Germany). We hereby kept to previously used stimulation durations (i.e., 20 min) to ensure maximal effects and hypothesised that prolonged stimulation effects would also support the training outcomes of tasks accomplished immediately after the end of the stimulation (Nitsche and Paulus, 2000; Elyamany et al., 2021). We therefore combined online and offline training within one session to also ensure a sufficient length of cognitive training. Sessions twice weekly over a period of 5 weeks were chosen as this corresponds to a typical clinical pattern for the administration of long-term interventions. Twice weekly sessions could be easily implemented in a routine setting and would be more feasible for participants than for example daily sessions. The sessions were performed in groups of three to six participants. The anode was placed over the left dorsolateral prefrontal cortex [5×7 cm, F3 according to the 10–20 EEG system (Klem et al., 1999)], the cathode (10×10 cm) was placed over the right supraorbital area (orientation as indicated in Figure 2). The ramping up/down time was 15 s in all stimulation groups and the stimulation setup allowed double blinding.

During the CCT participants trained processing speed, selective and divided attention, and executive functions (spatial working memory, inhibitory control) with the “CogniPlus” software (Schuhfried GmbH, Mödling, Austria). The tasks were displayed on 22-Inch desktop screens and the answers logged via simplified keyboards provided by the software company. During the stimulation participants trained either selective or divided attention. After the attention task, the stimulation electrodes were removed during a short break of approximately 10 min. In the second part of the session the participants performed two out of three tasks to train either spatial working memory, executive functions (inhibitory control) or speed of processing for 15 min each.

The study design is shown in Figure 2.

### 2.4. Statistical analyses

In the original study (Krebs et al., 2021) we found beneficial effects of tDCS in participants with low MoCA scores and no interaction between age and stimulation (Krebs et al., 2021) using linear mixed models. In the present study, we adopted a different analysis method, following recommendations from a study investigating which dependent and independent variables in a linear regression are best suited to predict the success of a CCT (Mattes and Roheger, 2020). According to Mattes and Roheger (2020) the best model includes baseline performance as one predictor, in combination with an interaction term between treatment outcome and predictor of interest (e.g., age). As outcome, the absolute change score is a valid choice (Mattes and Roheger, 2020). In the present study, we included the composite score at baseline in all models to address potential pre-existing differences in cognition. Values for age and years of education were mean centered and standardized. To further investigate significant interaction effects, we addressed different scores in age and years of education in further regression models (mean age ± 1 SD for younger and older age; mean years of education ±1 SD for few and many years of education). These further analyses allowed us to investigate interaction terms with a specific focus on certain factor levels. For descriptive statistics, we separated the sample according to age tertiles (youngest-old, middle-old, oldest-old) and performed Kruskal-Wallis tests for investigating differences between all tertiles or *t*-tests when comparing only lowest and highest tertiles. To explore potential associations between age, sex, and years of education we performed correlation analyses before the linear regression models. Treatment outcome in the linear regression models was the composite score difference, i.e., post-intervention composite score – pre-intervention composite score. To confirm the previously reported non-significant interaction between stimulation technique and cognitive improvement through the CCT (Krebs et al., 2021), we repeated this previous analysis using the method described above. Additionally, we repeated the models including years of education with the total score of the cognitive reserve questionnaire. Using the novel analysis approach, we confirmed the non-significant results including education as moderating factor (Supplementary material). To explore the interaction between years of education, sex or age and stimulation in the present study, we first performed regression models investigating two-way interactions. Given that there might exist interactions in between moderating factors, we also analysed three-way interactions (i.e., two moderating factors plus stimulation) in a next step. Interaction terms which showed a tendency towards significance (i.e., *p*-values between 0.05 and 0.10) were further analysed with additional regression models corresponding to those for significant interactions. As our analyses were explorative, we did not correct *p*-values for the number of performed regression models.

## 3. Results

First, we investigated differences between age groups. The age groups did not differ in years of education or sex, however, they differed significantly in baseline composite score and composite difference scores (Table 1). The overall mean score from the Montreal Cognitive Assessment was 26.31 (SD ± 2.62, min = 21, max = 30), which is above the cut-off score of 22 for cognitive disorders (Freitas et al., 2012). There were no correlations between age and years of education (*r* = −0.07, *p* = 0.58) or age and sex (*r* = 12, *p* = 0.36). On average, males had more years of education (mean: 16 years) than females (13.57 years) (*t* = 2.85, *p* = 0.006).

**Table 1:** Descriptive statistics of different age groups.

	Youngest – Old (*n* = 20)	Middle – Old (*n* = 20)	Oldest – Old (*n* = 19)	*p*-value
Age (years)	65.15 (± 2.37)	71.65 (± 1.35)	78.74 (± 3.30)	**<0.001**^**a**^
Years of education	14.60 (± 3.76)	15.50 (± 3.40)	14.42 (± 3.37)	0.47^**a**^
Sex	10 males	12 males	9 males	0.71^**a**^
Baseline composite score	0.20 (± 0.54)	0.15 (± 0.57)	−0.38 (± 0.57)	**<0.001**^**a**^
Difference score	0.23 (± 0.20)	0.11 (± 0.25)	0.33 (± 0.33)	**0.03**^**a**^
MoCA score	27.2 (2.26)	26.3 (2.64)	25.4 (2.75)	0.09^a^

Mean values and standard deviations are reported. sham, sham stimulation; tDCS, transcranial direct current stimulation; tACS, transcranial alternating current stimulation; MoCA, Montreal cognitive assessment; *n*, number of participants; ^a^Kruskal-Wallis test, significant values (*p*<.05) are bold.

Overall, a paired *t*-test showed that the cognitive intervention was successful in improving the composite score regardless of the stimulation group (*t* = −6.18, *p* < 0.001). However, the linear regression model did not show any significant effect of stimulation group on composite score difference (*F*_(55,2)_ = 6.04, *p* = 0.15) confirming the previously reported results.

Regardless of the respective stimulation group, participants with fewer years of education (lowest tertile) improved more in the cognitive composite score than participants with more years of education (highest tertile) (*t*_(30.68)_ = 2.57, *p* = 0.02). There was no similar effect when comparing the lowest and highest tertiles in age (*t*_(30.68)_ = −1.13, *p* = 0.27) or when comparing females and males in the complete sample (*t*_(56.02)_ = −0.98, *p* = 0.33).

The regression model investigating the association between age and stimulation did not show a significant interaction (*F*_(6,52)_ = 2.38, *p* = 0.10). Further regression analysis showed a significant tDCS effect in oldest participants compared to sham stimulation (*t* = 2.79, *p* < 0.001). The other models did not show an interaction between sex and stimulation (*F*_(6,52)_ = 1.87, *p* = 0.16) or years of education and stimulation.

**Figure 3:**
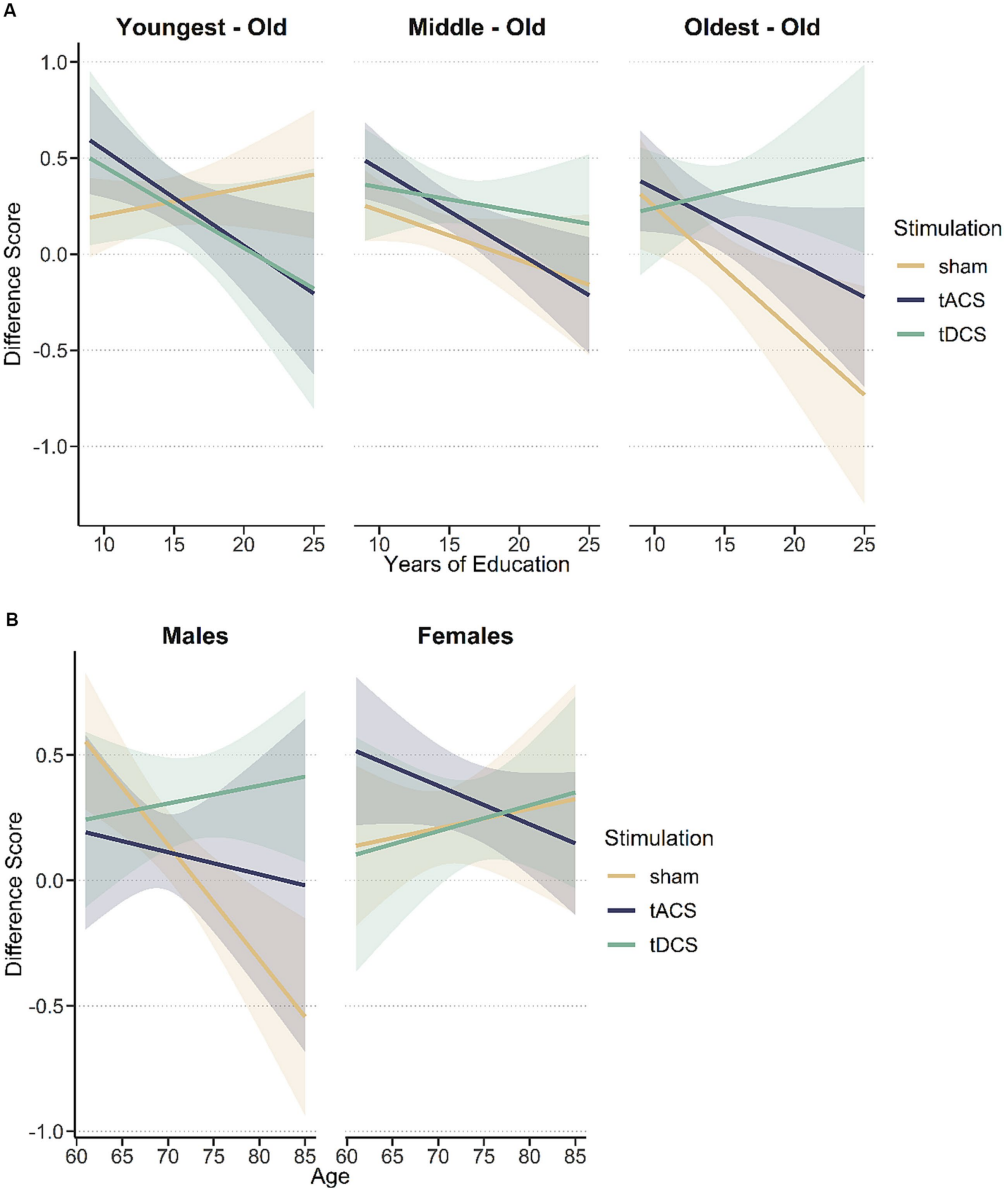
**(A)** Significant three-way interaction between age, years of education, and stimulation on composite score differences. There is a positive tDCS effect in oldest adults while in youngest adults tACS might have some beneficial effects. **(B)** There was a trend (*p* = 0.9) for a significant interaction between sex, age and stimulation on composite score differences. TDCS might be beneficial in oldest males, while tACS seems to support the efficacy of the cognitive training in youngest females.

The regression model including the three-way-interaction between years of education*age*stimulation showed a significant interaction (*F*_(12,46)_ = 5.53, *p* = 0.007). Compared to sham, tDCS was most beneficial in the oldest and highest educated participants (*β* = 68, *t* = 4.01, *p* < 0.001) while in the youngest individuals with fewest years of education tACS showed a tendency for beneficial effects (*β* = 25, *t* = 1.95, *p* = 0.058) (Figure 3A). The model including the three-way-interaction between years of education*sex*stimulation did not show a significant interaction (*F*_(12,46)_ = 2.12, *p* = 0.13), while the model including age*sex*stimulation showed a trend towards significance (*F*_(12,46)_ = 2.56, *p* = 0.09). When data was separated by sex and the regression models were repeated, younger and average aged females showed a significant tACS effect (younger females: *β* = 0.36, *t* = 3.10, *p* < 0.05; average aged females: *β* = 0.17, *t* = 2.11, *p* < 0.05) and a tDCS effect was present in older and average aged males (average aged males: *β* = 0.32, *t* = 2.65, *p* = 0.01; older males: *β* = 0.67, *t* = 4.08, *p* < 0.001) compared to sham stimulation (Figure 3B).

There was no difference in side effects between stimulation groups and blinding was successful [see Krebs et al. (2021) for details].

## 4. Discussion

The aim of our analyses was to investigate the moderating effects of age, years of education, and sex on a cognitive intervention combining CCT with different tES protocols (tDCS, tACS, and sham). While there were no interactions between each of the single factors and the stimulation group in two-way interactions, we found a significant three-way interaction between years of education, age and stimulation. Furthermore, there was a tendency towards significance in the interaction between sex, age, and stimulation.

Our examinations of the three-way interaction (years of education, age and stimulation) showed, that especially older participants with more years of education benefitted from tDCS, while in young adults with fewer years of education tACS seemed more promising to augment the effect of a CCT. Regarding tACS, beneficial effects on long term memory were observed after high-definition stimulation over the left DLPFC with gamma tACS up to 1 month. Theta tACS over the left inferior parietal lobe showed beneficial effects in a working memory task in older adults in the same study. Interestingly, this tACS effect was strongest in participants with low cognitive performance, as assessed with the MoCA (Grover et al., 2022). The design of the present study and those of Grover et al. (2022) are different in many ways (e.g., stimulation site, electrode type, stimulation schedule), which might account for the differing findings. Our finding of a tendency towards significance in younger participants with fewer years of education should be confirmed in further studies. Regarding the efficacy of tDCS, we suggested in previous research that tDCS is likely not beneficial when brain functions are optimal but rather becomes effective when a crucial level of cognitive decline is reached (Krebs et al., 2020, 2021). In the oldest adults in the sham group the improvement in the composite score difference became smaller or even negative with more years of education while the opposite pattern was visible in the tDCS group. It is possible, that in oldest participants more years of education led to optimized cognitive processes or implicit strategies to solve cognitive tasks which cannot be further improved through CCT itself. However, it is possible that the synergistic effects of CCT and brain stimulation allow a further increase in performance through more efficient cognitive processes. During anodal tDCS a certain brain region is targeted which is expected to become more active through the stimulation (Polanía et al., 2018). In the present study we targeted the left DLPFC, which is thought to be a hub for executive control processes and a brain area that is widely connected to various other brain regions such as the parietal lobe or the hippocampus (Hertrich et al., 2021; Smucny et al., 2022). Especially the left DLPFC has been stimulated successfully to increase performance in previous studies targeting different cognitive domains (Mancuso et al., 2016; de Lara et al., 2017). Regarding our findings, it is possible that tDCS increased cognitive control processes. Those improved control processes could positively affect brain networks or task solving strategies, which are predominantly used by individuals with more years of education and lead to larger training benefits. The positive effect of tDCS over the DLFPC in older adults with more years of education was also reported in another study using a working memory paradigm. The authors assumed that this result is caused by different strategies in higher educated participants resulting in a better recruitment of the prefrontal cortex (Berryhill and Jones, 2012). One study (Assecondi et al., 2022) even reported that younger-old with higher baseline working memory capacity performed significantly better during working memory training without concurrent tDCS. Although our results numerically pointed towards the same direction for the youngest-old with high education, we could not confirm this previous finding statistically.

Interestingly, when scores from the cognitive reserve questionnaire were used in the linear regression model instead of years of education, there was no significant interaction. Both measures correlate significantly in our data (*r* = 0.50, *p* < 0.001) and years of education can be seen as a proxy of cognitive reserve (Mungas et al., 2018). One difference between both measures is, that the total score of the cognitive reserve questionnaire also includes subscales which address leisure time activities as well as working activity. Additionally, the scores calculated for the respective subscales are based on a formula and do not correspond to the sum of years. It is possible, that for identifying the moderating factors of non-invasive brain stimulation in healthy older adults an unprocessed proxy like number of years better represents the neural substrate of cognitive reserve than a more complex measure like questionnaire scores.

Because the three-way interaction including stimulation, sex, and age showed a trend towards significance, we performed further analyses which showed that males benefit more from tDCS if they are older. Therefore, it is possible that also the beneficial effect of tDCS in the previously reported interaction (stimulation, years of education, and age) was mainly driven by oldest males with many years of education. As there was not enough data to perform additional statistical analyses when the sample was split according to age tertiles, we inspected descriptive statistics to estimate if this assumption might be true. Actually, in the oldest-old tDCS group males improved more but had fewer years of education (composite score difference: 0.51, years of education: 15.3 years) than in the sham group (composite score difference: −0.13, years of education: 18 years). Therefore, we assume that more years of education support tDCS effects regardless of sex. For youngest females in our sample, it seems that tACS led to more benefits of the CCT. This is in line with previous research which found a positive effect of tACS in young adult females (Pahor and Jaušovec, 2016).

While the sample size of 59 participants seems appropriate, it provides only limited data for subgroups, especially if multiple attributes are combined. Additionally, the trend for positive tACS effects in youngest adults with low education should be further investigated in young samples in a cognitive intervention. Given the explorative purpose of our analyses, we did not correct *p*-values for the number of performed regression models, which might lead to an overestimation of our results. As we aimed to include also participants with few years of education and across a considerably broad age range of 25 years, we did not define MoCA scores below a certain cut-off score as exclusion criteria. Therefore, we included three participant which are healthy based on self-reports, but the MoCA indicates that some cognitive impairment might be present.

In conclusion, there exist complex interactions between individual characteristics affecting the outcome of CCT combined with tES. Our findings indicate that tDCS might be most beneficial in oldest and highest educated individuals or males regardless of years of education. In youngest females in our sample, it seems that the combination of a CCT and tACS might lead to improvements in cognitive outcomes. These results emphasize the importance of further investigating and considering sex, age, and education as moderating factors in the development of individualized stimulation protocols.

## Data availability statement

The data that support the findings of this study is available from the corresponding author upon reasonable request.

## Ethics statement

The study was approved by the Kantonale Ethikkommission Bern, Switzerland (Nr. 2017-02056) and was conducted in accordance with the local legislation and institutional requirements. Participants gave their written informed consent before study onset.

## Author contributions

SK, CK, JP, and A-KB designed the research project. CK was responsible for data collection with support from EB. CK performed the data analysis. CK and A-KB drafted the manuscript. All authors contributed to the article and approved the submitted version.
