# Supplementary material for: The moderating effects of sex, age, and education on the outcome of combined cognitive training and transcranial electrical stimulation in older adults
Source: Front Psychol. 2023 Sep 22;14:1243099. doi: 10.3389/fpsyg.2023.1243099 (PMC10556861; doi:10.3389/fpsyg.2023.1243099)
Supplement: Supplementary file 1 [file Table_1.DOCX]

# Supplementary material

Participants in the study performed the cognitive reserve index questionnaire (CRIq; Nucci et al., 2012). We repeated the linear regression model from the main manuscript with the CRIq totalscores instead of years of education.

The CRIq total score correlated significantly with years of education (*r* = 0.50, *p* < .001).

The linear regression model did not show an interaction between CRIq score and stimulation on the

composite difference score (*F*_(52,2)_ = 0.03, *p* = .97). In the more complex regression models there were no significant three-way interactions (stimulation*CRIq score*YoE: *F*_(46,2)_ = 0.13, *p* = .88; stimulation*CRIq score*Age: *F*_(46,2)_ = 0.97, *p* = .38). Due to the lack of significant results or

tendencies towards significance no further analyses including the CRIq scores were performed.
